# Overdose responses among rural people who use drugs: A multi-regional qualitative study

**DOI:** 10.1186/s12954-024-01007-9

**Published:** 2024-05-31

**Authors:** Robin Baker, Rob J Fredericksen, Abby E Rudolph, Thomas J Stopka, Suzan M Walters, Monica Fadanelli, Rebecca S Bolinski, Adams L Sibley, Erin Stack, Heidi M Crane, P Todd Korthuis, David W Seal

**Affiliations:** 1 OHSU-PSU School of Public Health, Portland, OR USA; 2 Department of Medicine, University of Washington, Seattle, WA USA; 3 Department of Epidemiology and Biostatistics, Temple University College of Public Health, Philadelphia, PA USA; 4 Department of Public Health and Community Medicine, Tufts University School of Medicine, Boston, MA USA; 5 Department of Epidemiology, School of Global Public Health, New York University, New York, NY USA; 6 Rollins School of Public Health, Emory University, Atlanta, GA USA; 7 Southern Illinois University, Carbondale, IL USA; 8 The University of North Carolina at Chapel Hill, Chapel Hill, NC USA; 9 Comagine Health, Portland, OR USA; 10 University of Washington, Seattle, WA USA; 11 School of Medicine, Oregon Health & Science University, Portland, OR USA; 12 Tulane University School of Public Health and Tropical Medicine, New Orleans, LA USA; 13 Learning Design & Innovation, 37 Dewey Field Rd, Suite 201-205, 03755 Hanover, NH USA

**Keywords:** Overdose, Opioid use disorder, Qualitative research, Harm reduction, Rural communities, Peer response

## Abstract

**Background:**

Efforts to distribute naloxone have equipped more people with the ability to reverse opioid overdoses but people who use drugs are often reluctant to call 911 due to concerns for legal repercussions. Rural communities face unique challenges in reducing overdose deaths compared to urban communities, including limited access to harm reduction services as well as greater concerns about stigma and privacy.

**Methods:**

The Rural Opioid Initiative was funded in 2017 to better understand the health-related harms associated with the opioid crisis in rural US communities and consists of eight studies spanning ten states and 65 counties. Each study conducted semi-structured qualitative interviews with people who use drugs to understand contextual factors influencing drug use and health behaviors. We analyzed qualitative data from seven studies with data available at the time of analysis to understand peer response to overdose.

**Results:**

Of the 304 participants interviewed, 55% were men, 70% were white, 80% reported current injection drug use, and 60% reported methamphetamine use. Similar to what has been found in studies focused on urban settings, people who use drugs in rural communities use a range of strategies to reverse overdoses, including non-evidence-based approaches. Several reported that multiple doses of naloxone are needed to reverse overdose. Three themes emerged around the willingness to call 911, including (1) hesitancy to call 911 for fear of legal consequences, (2) negative perceptions or experiences with law enforcement officers, and (3) efforts to obtain medical intervention while avoiding identification/law enforcement involvement.

**Conclusion:**

People who use drugs employ multiple strategies to attempt overdose reversal, including non-evidence-based approaches. Greater education about the most effective and least harmful strategies is needed. Reluctance to call 911 is rooted in concerns about potential legal consequences as well as perceptions about law enforcement officers, which may be heightened in rural communities where people who use drugs are more easily identified by law enforcement. People who use drugs will go to great strides to connect their peers to needed medical services, suggesting that comprehensive interventions to reduce interactions with law enforcement officers and eliminate legal consequences for reporting overdoses are critical.

## Introduction

Opioid-related overdose rates have steadily increased since 2000. Provisional data from the CDC’s National Center for Health Statistics indicate that there were an estimated 77,766 opioid overdose deaths in the US during the 12-month period ending in December 2021, an increase of 12.6% from the 69,061 deaths during the 12-month period ending in December 2020 [1]. Of the 77,766 opioid overdose deaths reported in 2021, almost 88% involved synthetic opioids, primarily fentanyl [1]. While rates of overdose deaths are higher in urban settings, rural communities face unique challenges related to opioid use disorders due to reduced access and greater distances to behavioral health services and providers, limited public transportation, greater experience with stigma, and more concerns about privacy [2–5]. People who use drugs are well positioned to serve as first responders in the event of overdose [6, 7]. However, research also suggests that most people who use drugs do not call 911 when witnessing an overdose [8, 9]. Common reasons include fear of law enforcement officer involvement [8, 10] and the belief that emergency medical services (EMS) are not needed [8]. One study found that overdose location may be an important factor, with people who use drugs more likely to call 911 when overdoses occur in public settings versus private residences or locations with active drug dealing and open drug use [11].

As of 2021, 47 states and Washington, D.C., have Good Samaritan laws (GSL) in place, which aim to protect bystanders from arrest or conviction when calling 911 to report an overdose [12]. Studies demonstrate that GSL have addressed some of the obstacles associated with calling 911 to report overdoses; however, limitations of GSL protections exist [13–15]. These include the narrow scope of protection that some GSL provide, a lack of awareness and understanding of the law among both people who use drugs and law enforcement officers, perceptions of and experiences among people who use drug with law enforcement officers disregarding the law [10, 13–15].

Efforts to increase the capacity of people who use drugs to respond appropriately to overdoses include overdose education and naloxone distribution (OEND) programs [16]. Commonly recommended strategies to rescue people from opioid overdoses include sternum rubs, rescue breathing, naloxone administration, placing the person in the recovery position, and calling 911 [16]. Calling 911 is important because once the reversal effects of naloxone wear off, overdoses can reoccur, particularly as fentanyl and its analogs become the leading cause of overdose [17]. The increasing availability of naloxone through pharmacies, harm reduction agencies, health departments, and other community-based programs can equip people who use drugs with the tools needed to reverse opioid overdoses [16, 18]. While several studies have demonstrated that distributing naloxone and training people who use drugs to administer naloxone reduces overdose mortality in communities [19–21], reluctance or hesitancy to call 911 persists. Most studies on overdose responses have focused on urban settings [10, 22, 23].

Studies focused on rural communities have similarly found that rural people who use drugs fear the legal consequences of reporting overdose, even in states with GSL [24]. As with urban communities, people who use drugs often report experiencing stigma related to drug use in rural communities [11, 25–27]. In addition, rural communities also have limited access to substance use and harm reduction resources [3, 5] and a larger proportion of overdose deaths involving psychostimulants (e.g., cocaine, methamphetamine) [28]. Limited access to OEND programs and increased rates of psychostimulant-involved overdose deaths is particularly concerning as previous studies have found that some people who use drugs mistakenly believe that psychostimulants can reverse opioid overdoses [29, 30]. These unique challenges and factors may influence how rural people who use drugs respond to overdoses. This qualitative study builds on the existing literature by characterizing overdose response patterns and the interplay of factors influencing EMS involvement in a geographically diverse multi-regional U.S. sample of people who use drugs.

## Materials and methods

The Rural Opioid Initiative (ROI) was funded in 2017 to better understand the health-related harms associated with the opioid crisis in rural parts of the United States (http://ruralopioidinitiative.org/). ROI consists of eight study regions spanning ten states and 65 counties [31, 32]. The initiative included qualitative and quantitative data collection, with harmonized data collection instruments, as well as epidemiologic, policy, and legal scans [33, 34]. Each study region conducted semi-structured individual qualitative interviews with people who use drugs to better understand contextual factors, life history, and circumstances influencing drug use and health behavior.

### Study settings

This manuscript reports on findings from seven study regions: Illinois (IL), Kentucky (KY), North Carolina (NC), Northern New England (NE; Vermont, New Hampshire, Massachusetts), Ohio (OH), Oregon (OR), and Wisconsin (WI). West Virginia was excluded due to lack of complete data at the time of analysis. Each study region focused on rural communities with high rates of hepatitis C virus (HCV) infection and opioid overdose fatalities. All study regions included counties that were identified in 2016 by the Centers for Disease Control and Prevention (CDC) as experiencing or at risk of experiencing increases in HCV infection due to injection drug use and several included counties that were identified as among the most vulnerable counties in the US for HIV/HCV outbreaks linked to injection drug use, including Illinois, Kentucky, North Carolina, Northern New England, and Ohio [35]. Among the nine states included in this manuscript, all but Oregon had higher opioid overdose death rates than the national opioid overdose death rates in 2018 and 2020, and most saw an increase in opioid overdose death rates from 2018 to 2020 [36]. In 2019 and 2020, three of the nine states had higher estimated rates of past-year methamphetamine use among individuals aged 18 years and older compared to the national estimate of under 1%; Oregon had nearly double with over 2% [37]. Six states had higher estimates of past year cocaine use among individuals aged 18 years and older compared to the national estimate of just over 2%; New Hampshire and Vermont had almost 3% [37].

### Interview guide development

The interview guide was developed collaboratively by ROI researchers with expertise in qualitative methods, representing all ROI study regions, who comprised the ROI Qualitative Methods Workgroup. The development of a standardized interview guide ensured uniformity of primary content across studies. The interviews included but were not limited to topics related to illegal opioid and other drug use and access to and use of harm reduction, substance use, mental health, and health care services. Of particular interest for this study were questions related to observations of overdose. Participants were asked, “Tell me about your most significant experience with someone else overdosing?” Common follow-up questions probed for the overdose location; other people’s responses; the participant’s response; whether 911 was called; who arrived first (i.e., law enforcement or EMS); whether the person went to the hospital; if naloxone was used, and if so, by whom; and which drugs were involved. Probes varied across studies to enable investigators within each study region to follow-up on issues specific and relevant to their location and communities. Each study received approval from a local institutional review board and participant privacy was protected by a federal certificate of confidentiality.

### Participant recruitment and data collection

Between 2018 and early 2020, we recruited people who used drugs to participate in interviews ranging from 60 to 90 min. Across all studies, qualitative interview participants had to reside in the study area and be at least 18 years old. Most sites required participants to report opioid use “to get high” or injection drug use in the past 30 days. Other eligibility criteria varied across sites due to regional differences in drug use and drug-related harms. For example, Ohio specifically recruited people who recently transitioned to injection drug use and women with experiences of neonatal opioid withdrawal, North Carolina focused on people who injected painkillers or heroin, and Wisconsin recruited people who injected opioids in the past month. All studies recruited participants from community-based programs and in some cases used street outreach. Trained ualitative researchers, many of whom had extensive training and experience, conducted the interviews, which were digitally recorded and transcribed verbatim. Participants were consented per the study IRB protocols and compensated with an incentive ranging from $25–50 depending on the study.

### Analysis

Identifying information was redacted from the transcripts. Each transcript was assigned a unique identification number and uploaded to a qualitative software program for data management, coding, and analyses (Dedoose, Los Angeles, CA). The Qualitative Core of the University of Washington ROI Data Coordinating Center conducted preliminary coding to categorize data by interview topic areas and lines of inquiry to facilitate retrieval of relevant data from the larger multi-study dataset. Upon retrieval of the data related to overdose in the preliminary coding round, we developed a data-driven thematic coding scheme that was iteratively refined by the writing team following principles of grounded theory analysis [38, 39]. We analyzed the data to identify recurring themes, focusing on better understanding overdose experiences and responses. The writing team held regular meetings to discuss new thematic categories or codes that emerged in the data and to ensure consistency in coding/thematic definitions and application. This iterative process continued until we achieved thematic saturation and stabilized the organization of the findings. Nonverbal utterances were removed to improve the readability of the quotes.

## Results

A total of 304 participants completed a qualitative interview. The mean age was 36 years and 55% of the participants were men. Among the 169 for whom race data were available, 70% were white. 32% had a high school diploma or GED, 20% had some college, and 18% had less than a high school diploma or GED. 80% reported current injection drug use and 60% reported methamphetamine use. See Table 1 for demographic characteristics of interview participants, by study.

**Table 1:** ROI Interview Participant Demographic Characteristics

Interviewees		Total	Illinois	Kentucky	North Carolina	New England	Ohio	Oregon	Wisconsin
		304 (100%)	22 (7%)	57 (19%)	65 (21%)	22 (7%)	26 (9%)	52 (17%)	60 (20%)
Male^1^		169 (55%)	14 (64%)	35 (61%)	34 (52%)	10 (45%)	15 (58%)	28^1^ (54%)	33 (54%)
Average age		36	37	35	36	33	37	39	35
Race									
	White	213 (70%)	20 (91%)	56 (98%)	15 (23%)	15 (68%)	*not asked*	49 (94%)	58 (97%)
	Black	2 (1%)	2 (9%)	0 (0%)	0 (0%)	0 (0%)	--	0 (0%)	0 (0%)
	Native American	8 (3%)	0 (0%)	0 (0%)	5 (8%)	1 (5%)	--	1 (2%)	1 (2%)
	Mixed race	5 (2%)	0 (0%)	0 (0%)	2 (3%)	0 (0%)	--	2 (4%)	1 (2%)
	Other	1 (0%)	0 (0%)	1 (2%)	0 (0%)	0 (0%)	--	0 (0%)	0 (0%)
	Not given/Not asked^2^	75 (25%)	0 (0%)	0 (0%)	43^2^ (66%)	6^2^ (27%)	26^2^ (100%)	0 (0%)	0 (0%)
**Interviewees**		**304 (100%)**	**22 (6%)**	**57 (16%)**	**65 (19%)**	**22 (6%)**	**26 (7%)**	**52 (15%)**	**60 (17%)**
Substance use									
	Ever IDU	288 (83%)	20 (91%)	57 (100%)	65 (100%)	21 (95%)	17 (65%)	52 (100%)	56 (93%)
	Current IDU	279 (80%)	19 (86%)	57 (100%)	65 (100%)	18 (82%)	15 (58%)	50 (96%)	55 (92%)
	*Heroin*	195 (56%)	8 (36%)	30 (53%)	51 (78%)	18 (82%)	13 (50%)	41 (79%)	34 (57%)
	*Fentanyl*	73 (21%)	1 (5%)	17 (30%)	26 (40%)	14 (64%)	3 (12%)	6 (12%)	6 (10%)
	*Methamphetamine*	210 (60%)	14 (64%)	37 (65%)	61 (94%)	4 (18%)	6 (23%)	47 (90%)	41 (68%)
	*Other*	193 (55%)	4 (18%)	54 (95%)	61 (94%)	15 (68%)	10 (38%)	21 (40%)	28 (47%)
**Interviewees**		**304 (100%)**	**22 (7%)**	**57 (19%)**	**65 (21%)**	**22 (7%)**	**26 (9%)**	**52 (17%)**	**60 (20%)**
Education									
	< High school	56 (18%)	1 (5%)	21 (37%)	13 (20%)	3 (14%)	7 (27%)	*not asked*	11 (18%)
	H.S. or GED	97 (32%)	11 (50%)	22 (39%)	21 (32%)	9 (41%)	11 (42%)	--	23 (38%)
	Some college	61 (20%)	7 (32%)	6 (11%)	20 (31%)	3 (14%)	6 (23%)	--	19 (32%)
	Assoc/trade deg	16 (5%)	3 (14%)	8 (14%)	0 (0%)	1 (5%)	0 (0%)	--	4 (7%)
	>= B.A.	12 (4%)	0 (0%)	0 (0%)	8 (12%)	0 (0%)	1 (4%)	--	3 (5%)
	Not answered	62 (20%)	0 (0%)	0 (0%)	3 (5%)	6 (27%)	1 (4%)	52 (100%)	0 (0%)

1. One non-male interviewee from Oregon identifies as “Neither”.

2. Some NC and NE interviewees did not give their race. Ohio did not ask interviewees their race.

Rural people who use drugs in our study reported using multiple strategies, and often in combination, to attempt overdose reversal. Participants reported sternum rubs, rescue breaths, naloxone, application of ice or cold water, CPR, chest compressions, and inflicting pain by slapping or hitting their peers.

He was just completely out. It was really crazy. We had to jump out and pull him out of the car and slap him a little bit and throw cold water on him. (28-year-old man, OR)

Chest compressions…anything I could think of to do…blow in their mouth…CPR…I carried a 180-pound man into a shower before and threw him in cold water, and that’s what got him out. (23-year-old woman, NE)

Of particular concern, some participants also reported using psychostimulants to reverse overdose (i.e., methamphetamine in OR, OH, IL, and NC; crack or cocaine in NE). In many of these cases, participants indicated that they used psychostimulants after other overdose reversal strategies did not work.

I tried giving him CPR and it didn’t work…I took his needles and put methamphetamine in it and shot it in his hand. Within about, probably 20 to 30 s, he jumped up and ran out the door. I know for a fact that if I hadn’t shot that speed into him, his heart would have stopped, because [his] face [was] blue, lips white…and [he was] breathing real funny and puking on himself and stuff. (30-year-old man, OH)

Finally, while participants reported using naloxone to reverse overdoses, notably, several talked about the need for multiple doses of naloxone, sometimes as many as 3–7 doses.

We tried Narcan three times. It didn’t work. (Cries)…Hours passed and finally…I had to go ask for help, I didn’t know what to do. And they called the ambulance to come get her. We tried the cold shower, we tried everything…it didn’t work. (38-year-old woman, NE)

In addition, multiple themes emerged regarding the decision to call 911. These included (1) hesitancy to call 911 for fear of legal consequences, (2) negative perceptions of, or experiences with law enforcement officers, and (3) efforts to obtain medical intervention while avoiding identification/law enforcement involvement.

### Hesitancy to call 911

Among those participants who considered calling 911 to report witnessing an overdose, they shared conflicted feelings about calling 911 because they were concerned about the potential legal consequences. For example, many participants discussed their fear of losing child custody, getting arrested, or going to jail. Several participants also reported that they did not call 911 because they were respecting the wishes of the person who overdosed. In some cases, participants indicated that their peers made it known that they did not want 911 called if they overdosed. In other cases, participants indicated that they wanted to call 911 after they revived their peer, but the peer said no.

We probably should have [called 911], but we didn’t…we were scared of getting into trouble. We were doing drugs, which is really not an excuse. We should’ve called 911, but luckily, I was able to bring her back. (29-year-old man, KY)

I’m a single mom, and my daughter was in the other room… I was afraid to go to jail… I would’ve called 911 if we couldn’t have gotten him to come through…but I did avoid it because I didn’t want to lose my kid and I didn’t want to go to jail for attempting murder. (36-year-old woman, WI)

I didn’t call 911 because he refused it. I told him I was going to call, but he refused. So I didn’t call. (26-year-old woman, KY)

### Perceptions of law enforcement officers and emergency medical services in small towns

A few participants reported perceptions of law enforcement or EMS that decreased their willingness to call 911. For example, one respondent noted that the local law enforcement officers are corrupt, while another noted that their local EMS is less likely to respond in a timely fashion for overdoses.

               The cops are shit…they treat people like they’re dirt, you know. (60-year-old woman, IL)

They [police] are involved in a lot of the stuff that goes on…Prostitution and stuff like that…I just don’t trust anyone down there. That is another reason why like I would be scared to call the law if someone overdosed. I mean, I would do it, I would, but I understand why people are scared because of that. (33-year-old woman, OH)

The few times that EMS has been called, they take their time. We’re told not to even tell them that it’s an overdose. Tell them there’s an emergency, but don’t tell them that, because they’ll take their time. Small towns are like that. It is a very who you know and who you are, and if you do drugs, you’re automatically same category as a murder. (38-year-old woman, NC)

### Obtaining medical intervention while avoiding identification and law enforcement involvement

Despite expressing a reluctance to call 911, several participants reported efforts to connect their peers with medical intervention while minimizing the chance of law enforcement involvement. These included driving to a public place before calling 911, driving the person to the hospital, or calling 911 and leaving the vicinity.

I called the ambulance and took off…I called them and said that there’s somebody sitting in there and I didn’t know what to do. I told them her name…told them she was alone in her house and she needs somebody to come and I was gone. (43-year-old woman, NC)

Her mother overdosed, and…the guy that owned the house, was like, ‘get him out of here.’ We’re going to call the cops and we’re going to call the ambulance. [They said, ] ‘Oh, no you’re not,’ so we carried this woman outside and put her in a car and I drove to a bar that was a block away and…I called the cops and I called the ambulance. (44-year-old man, WI)

## Discussion

Interviews with people who use drugs in rural communities revealed a range of barriers to accessing emergency care and a range of strategies to reverse overdoses, including naloxone, slapping or hitting, ice or cold water, psychostimulants, CPR, chest compressions, and rescue breathing. Complex and interconnected factors influenced whether and how people responded. Hesitancy to call 911 or to remain with the peer after calling 911 was common, largely due to fear of legal repercussions, particularly among those with prior criminal justice involvement. Another factor that influenced whether people called 911 was negative perceptions of or prior interactions with local law enforcement or EMS. As a result, some participants reported workarounds to connect their peers with medical services while reducing law enforcement involvement.

The use of multiple strategies demonstrates that rural people who use drugs proactively respond to reverse an overdose and persist if the first intervention is not successful. It is important to note that there are some variations in recommendations for overdose response. SAMHSA and the World Health Association recommend rescue-breathing, while the American Heart Association does not [40]. These differences in recommendations are, in part, based on differing perceptions of the ability of laypersons to correctly identify overdose, respiratory or cardiac arrest symptoms, and carry out the appropriate intervention [40]. However, some strategies reported by participants, such as inflicting pain and using ice or water, are not recommended and can either cause additional injury or delay the implementation of safer and potentially more effective overdose strategies [22]. The reported folk method of using methamphetamine, crack, and cocaine to reverse overdoses highlights persistent system failures in ensuring that people have access to comprehensive overdose reversal education and access to naloxone. In 2018, nearly three-quarters of all cocaine overdose deaths and half of all methamphetamine overdose deaths also involved opioids [41], and co-use is associated with an increase in the risk of overdose in rural communities [42]. Expanding OEND programs and partnering with trusted community-based organizations and people who use drugs in rural communities may be an effective way to disseminate evidence-based overdose strategies directly to people actively using drugs. Further research is needed to understand the underlying systemic issues contributing to the use of psychostimulants to reverse overdoses and to identify strategies to increase overdose reversal education and the distribution of naloxone.

Many participants reported that multiple doses of naloxone were often needed to reverse overdose. Several studies have found that higher doses of naloxone are needed to reverse overdose caused by synthetic opioids [43–45]. Given the rise in overdose deaths attributed to fentanyl and related analogs, further overdose reversal research is warranted while counseling people to continue dosing naloxone as needed and call 911 [46]. The method of administration may make a difference. While intranasal naloxone has become popular because of ease of administration and increased accessibility, a randomized clinical trial found that clients at a Canadian overdose prevention site who received injectable naloxone were significantly less likely to need an additional rescue dose than clients who received intranasal naloxone [47]. One possible strategy to explore is the distribution of injectable naloxone both to people who use drugs who are comfortable with injections and EMS personnel. This could provide a cost-effective method and facilitate more efficient single doses of naloxone with a decreased need for additional doses. It is also critical to increase the quantity of naloxone, regardless of the mode of administration, distributed to rural people who use drugs and EMS and wherever fentanyl or other analogs are prevalent. Given reports among participants that it sometimes takes more than three doses of naloxone to reverse overdose, it is important that people have access to multiple naloxone kits so that they are better equipped to respond to an overdose. In addition, in rural communities where there may be long driving distances between the overdose location and the closest hospital [48], multiple doses of naloxone may be necessary in case the reversal effects of naloxone wear off before the person is connected to EMS.

These findings highlight the impact that fear of law enforcement involvement has on responses among those witnessing overdose. Concerns about legal consequences inhibit the ability of rural people who use drugs to respond appropriately. These concerns may be compounded by the relative lack of anonymity in rural communities [49]. The likelihood that rural people who use drugs are well known and easily identified by law enforcement officers is substantially increased compared to urban communities due to tighter interconnected social networks, lower population density, and smaller population counts [26]. These concerns may be heightened in small towns, as some participants shared perceptions of their local law enforcement officers and EMS that decreased their willingness to call 911, such as the perception that GSL would not be enforced.

While 47 states and Washington D.C. have GSL, there is significant variation in the protections they offer [10]. For example, of the nine states included in our study, only Ohio offers protections against the arrest, charge, and conviction for possession of illegal substances, and it limits immunity to only two instances, and only three states (Illinois, Massachusetts, and Vermont) provide provisions that allow reporting an overdose to be considered a mitigating factor in sentencing with significant variation in which crimes are permitted for mitigation [50]. In Ohio, the parole board or court has the discretion to mitigate the penalty [50]. However, ROI participants reported fear of legal repercussions across all studies, regardless of the presence or absence of GSL or the scope of protections. More research partnering with rural people who use drugs and law enforcement officers regarding awareness of existing GSLs and understanding of the protections and limitations of the GSL across settings is needed. Given that several participants shared the perception that small-town law enforcement officers are corrupt, it is possible that even if law enforcement officers are aware of an existing GSL, people who use drugs in rural communities may worry that the law would not be enforced. Persistent fear of legal repercussions and limitations of GSL protections underscore the need to explore policies that decrease the threat of legal action for consequences of drug use. Given recent efforts in other countries, such as Canada [51–54], to prevent and reduce overdose death, additional research on interventions such as overdose prevention sites and safer supply on overdose outcomes is needed.

Finally, another strategy could include the development of dedicated overdose call-in centers or emergency phone lines, like those established for suicide prevention [55], and potentially modeled after the “never use alone” crisis hotline (https://neverusealone.com/) [56, 57]. This could eliminate or significantly reduce law enforcement involvement with overdoses and directly connect people with needed medical services for overdoses. This would necessitate infrastructure development to ensure consistent Wi-Fi and cellular service, particularly in rural US communities [58, 59].

Our findings demonstrate that there is need for greater access to existing evidence-based interventions such as syringe exchange and drug checking services, distribution of naloxone, and medications for opioid use disorders. Overdose prevention requires a multi-faceted approach that includes increasing overdose reversal education, eliminating legal consequences of reporting an overdose, and providing direct pathways for people who use drugs to access medical assistance. Integrating harm reduction principles into the design and execution of these strategies could empower people who use drugs, often the true first responders, to save lives and significantly reduce overdose.

### Limitations & strengths

The study’s large sample size, and its diversity in geography (9 states and 58 counties) and age, offers the most comprehensive qualitative data on overdose responses among people who use drugs known to date. That said, our sample is largely white and cisgender, and the limited racial and gender diversity may limit the transferability of our findings to other settings and populations. In addition, study differences in drugs used, mode of drug administration, recruitment criteria, and follow-up probing questions may have influenced variation in responses. For example, the prevalence of methamphetamine use in some study locations may have provoked the use of methamphetamine as an overdose reversal strategy. Furthermore, while GSLs were in effect in all states included in the study at the time of data collection, participants’ retrospective recalls of overdose experiences may include overdoses that predate these laws, and such past decisions around medical intervention may have occurred in circumstances where legal protections were not yet afforded. Finally, this sample of rural people who use drugs is comprised mostly of persons who are engaged in harm reduction, who may have experiences that are unique from their peers who are not connected to such services.

## Conclusions

People who use drugs are well-situated to serve as first responders to reduce overdose deaths. This study confirmed that like people who use drugs in urban communities, the responses when witnessing overdoses in rural communities may be impacted by widespread fear of legal consequences, which heavily influences decision-making regarding whether, how, and when to access emergency care. In the face of this fear, and in the urgency of the moment, people who use drugs employ a wide range of strategies to attempt overdose reversal. Greater education is needed to ensure that people are well-informed about the most effective and least harmful opioid overdose reversal strategies. Access to naloxone should be increased among people who use drugs and first responders, including EMS and fire and police departments, particularly in rural communities. However, it is critical to remove barriers that prevent people who experience an overdose from receiving appropriate medical care. One of the main barriers, hesitancy to call 911, is rooted in concerns about the potential legal consequences of law enforcement involvement. These concerns may be heightened in rural communities where people who use drugs may be more easily identified by law enforcement officers. Our findings demonstrate the great strides people who use drugs in rural communities will take to reverse overdoses and save lives as well as the need for comprehensive interventions that eliminate legal consequences and expand harm reduction strategies for responding to overdose.

## Data Availability

To respect the confidentiality of participants in this study, data is not publicly available. However, we welcome collaboration and encourage mentorship and the use of the ROI data stripped of all protected health information (PHI) to enable early investigators to address meaningful questions with support to help ensure their success. Additional information can be obtained at the ROI website: ruralopioidinitiative.org or by contacting the ROI DCC at ruralopioidinitiative@uw.edu. Follow the Rural Opioid Initiative on Twitter @ruralopioids.

## Abbreviations

GSL: Good Samaritan laws
EMS: Emergency medical services
OEND: Opioid Education and Naloxone Distribution
ROI: Rural Opioid Initiative
